# Pressure effect of the mechanical, electronics and thermodynamic properties of Mg–B compounds A first-principles investigations

**DOI:** 10.1038/s41598-021-85654-z

**Published:** 2021-03-17

**Authors:** GuoWei Zhang, Chao Xu, MingJie Wang, Ying Dong, FengEr Sun, XiaoYan Ren, Hong Xu, YuHong Zhao

**Affiliations:** 1 School of Materials Science and Engineering, North University of China, Taiyuan, 030051 Shanxi China; 2 Department of mechanical engineering, Taiyuan Institute of Technology, Taiyuan, 030008 Shanxi China

**Keywords:** Materials science, Physics

## Abstract

First principle calculations were performed to investigate the structural, mechanical, electronic properties, and thermodynamic properties of three binary Mg–B compounds under pressure, by using the first principle method. The results implied that the structural parameters and the mechanical properties of the Mg–B compounds without pressure are well matched with the obtainable theoretically simulated values and experimental data. The obtained pressure–volume and energy–volume revealed that the three Mg–B compounds were mechanically stable, and the volume variation decreases with an increase in the boron content. The shear and volume deformation resistance indicated that the elastic constant C_ij_ and bulk modulus B increased when the pressure increased up to 40 GPa, and that MgB_7_ had the strongest capacity to resist shear and volume deformation at zero pressure, which indicated the highest hardness. Meanwhile, MgB_4_ exhibited a ductility transformation behaviour at 30 GPa, and MgB_2_ and MgB_7_ displayed a brittle nature under all the considered pressure conditions. The anisotropy of the three Mg–B compounds under pressure were arranged as follows: MgB_4_ > MgB_2_ > MgB_7_. Moreover, the total density of states varied slightly and decreased with an increase in the pressure. The Debye temperature Θ_D_ of the Mg–B compounds gradually increased with an increase in the pressure and the boron content. The temperature and pressure dependence of the heat capacity and the thermal expansion coefficient α were both obtained on the basis of Debye model under increased pressure from 0 to 40 GPa and increased temperatures. This paper brings a convenient understanding of the magnesium–boron alloys.

## Introduction

Magnesium boride alloys (MgB_2_, MgB_4_, and MgB_7_) as desirable compounds play an important role in many fields due to their remarkable conductivity, excellent ductility, and high hardness^1–3^. Usually, boron-rich magnesium alloys have excellent material characteristics such as mechanical properties and stability^4,5^. Moreover, MgB_2_ has been widely introduced into magnesium alloys for the reinforcement and grain refinement^6,7^, because of the chemical substitution and the crystal growth of substituted MgB_2_^8–11^. Therefore, increasing attention has been paid to investigate the magnesium boride alloys in many academic fields.

Superconductors of magnesium diboride were reported first by Akimitsu^12^ in 2001. Since then, magnesium boride systems have been extensively studied through theoretical simulations and experimental analyses^13–15^. The intermediate phases of Mg–B alloys, which include MgB_2_, MgB_4_, and MgB_7_, were found through the continued investigation of the Mg–B binary phase diagram using the CALPHAD method based on experimental data^16,17^. Furthermore, Brutti et al.^18^ studied the vaporisation behaviour of MgB_2_ and MgB_4_ by the Knudsen effusion-mass spectrometry technique. Wenzel et al.^19^ predicted the crystal system and the lattice parameters of Mg–B compounds by using the electron probe micro analysis (EPMA) and X-ray diffraction (XRD) analytical approaches. Moreover, Alapati et al.^4^ calculated the lattice parameters of Mg–B compounds using the first principle based on the density functional theory (DFT). The elastic constants, mechanical properties, bond structure, and electronic properties of MgB_7_ at 0 GPa were investigated by Ozisik^20^. Furthermore, the heat capacity and the thermal expansion of MgB_2_ at 0 GPa was predicted by Saengdeejing^21^. The thermodynamic properties of Mg–B compounds and Al–Mg–B films were also investigated by using ab initio calculations and CALPHAD methods^22,23^. So far, the most effective method to obtain the hexagonal phase MgB_2_ is the high-pressure and high-temperature growth by using different kinds of solvents, and the external pressures and higher temperature may promote the reaction of Mg–B compounds^22^. Moreover, the crystal structure, electronic properties, thermodynamic properties, and mechanical properties of Mg–B compounds at different pressure and temperature have not been studied.

Assuredly, the above mentioned experimental studies have evidenced that the properties of Mg–B compounds can be calculated using DFT for establishing the trends of stability through the cohesive energies and the trends of charge transfers onto boron. Therefore, in the current article, the structural, mechanism, electronic, and anisotropic properties of MgB_2_, MgB_4_, and MgB_7_ under pressure from 0 to 40 GPa were investigated by using DFT calculation. The thermal expansion coefficient, Debye temperature, heat capacity, and other thermodynamic properties were theoretically studied for determining the pressure and temperature dependence of Mg–B compounds.

## Computational methodology

In this study, all the calculated results were obtained by using the first-principle method through the Vienna ab initio simulation package (VASP)^24^ codes. PBE (Perdew–Burke–Ernzerhof)^25^ in GGA (generalized gradient approximation) was performed to expound the exchange-correction function^26^ and calculate the self-consistent electronic density. All the calculations in the current study were considered Mg 3p^6^3s^2^ and B 2s^2^2p^1^ as the valence electrons. To obtain an accurate calculated results, the cut-off energy E_cut_ was set to 500 eV. Moreover, the Brillouin-zone sampling mesh for the Monkhorst–Pack^27^ k-point for MgB_2_, MgB_4_, and MgB_7_ was set to 19 × 19 × 14, 9 × 11 × 7, and 8 × 8 × 8, respectively, due to the k-mesh was forced to be centred on the gamma point. Besides, the σ value of the first-order Methfessel–Paxton smearing was set to 0.2 eV, the convergence threshold of the self-consistent field was set to 1.0 × 10^−5^ eV/atom.

## Results and discussions

### Structural stability

**Figure 1:**
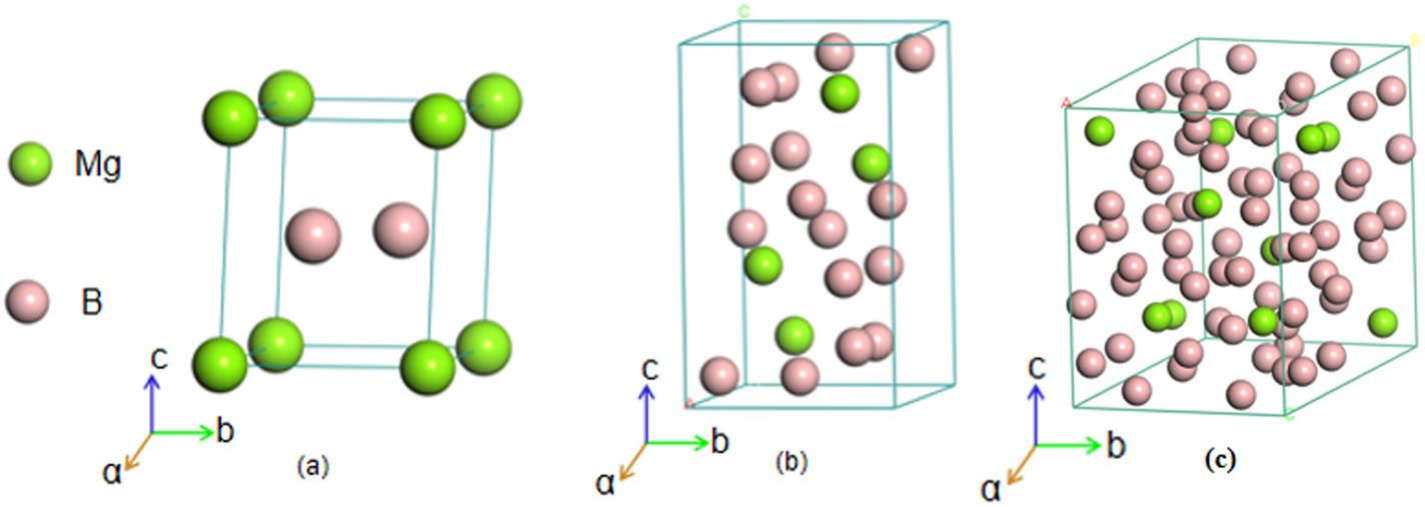
Optimised crystal structures of Mg–B compounds: (**a**) MgB_2_; (**b**) MgB_4_; (**c**) MgB_7_.

The optimised crystal texture of Mg–B compounds is shown in Fig. 1, and the corresponding calculated crystal parameters of the Mg–B compounds at 0 GPa are tabulated in Table 1^28–32^. As listed in Table 1, the simulated crystal structure parameters considered in this study matched well with the reported literature data from the experimental and theoretical calculations, which verified the reasonability of the Mg–B compound models.

**Table 1:** The simulated structure parameter of Mg–B compounds contained lattice constant (Å), bulk modulus B, and its derivative B_0_′.

Phase	Composition (at.% B)	Magnesium site	Space group	Unit cell lattice parameter (Å)			B (GPa)	B_0_′	Reference
				a	b	c			
MgB_2_	66.7	1a (0, 0, 0)	P6/mmm	3.07	3.07	3.53	151.7	3.54	This work
				3.08	3.08	3.52	157.0	3.50	Cal^28,29^
				3.08	3.08	3.52			Exp^30^
MgB_4_	80	4c (0.25, 0.546, 0.362)	Pnma	5.49	4.4	7.42	158.3	3.10	This work
				5.45	4.43	7.47			Cal^31^
				5.46	4.43	7.47			Exp^32^
MgB_7_	87.5	4c (0, 0.5, 0)	Imma	10.47	5.97	8.11	198.2	3.56	This work
				10.46	5.97	8.10	206.5		Cal^20^
				10.48	5.98	8.12	203.1		Exp^22^

**Figure 2:**
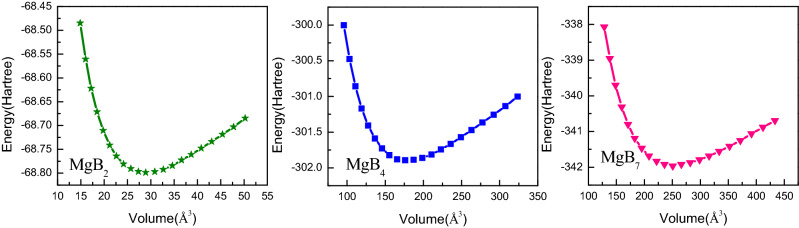
Variation between energy and volume of Mg–B compounds.

The energy–volume E(V) relation curves at zero absolute temperature were obtained using the first-principle method, as shown in Fig. 2. All the E(V) data were fitted to the Birch–Murnaghan model as follows^33^:

1 $$ E\left( V \right) = E_{0} + \frac{{9V_{0} B_{0} }}{16}\left\{ {\left[ {\left( {\frac{{V_{0} }}{V}} \right)^{\frac{2}{3}} - 1} \right]^{3} B_{0}^{{\prime}} + \left[ {\left( {\frac{{V_{0} }}{V}} \right)^{\frac{2}{3}} - 1} \right]^{2} \left[ {6 - 4\left( {\frac{{V_{0} }}{V}} \right)^{\frac{2}{3}} } \right]} \right\} $$

where B_0_ is the bulk modulus, B_0_′ is the first pressure derivative of the bulk modulus, and V_0_ is the equilibrium volume.

2 $$ B_{0} = - V\left( {\frac{dP}{{dV}}} \right)_{P = 0} = V_{0} \left( {\frac{{d^{2} E\left( V \right)}}{{d^{2} V}}} \right)_{{V_{0} }} \quad B_{0}^{{\prime}} = - \left( {\frac{dB}{{dP}}} \right)_{P = 0} $$

**Figure 3:**
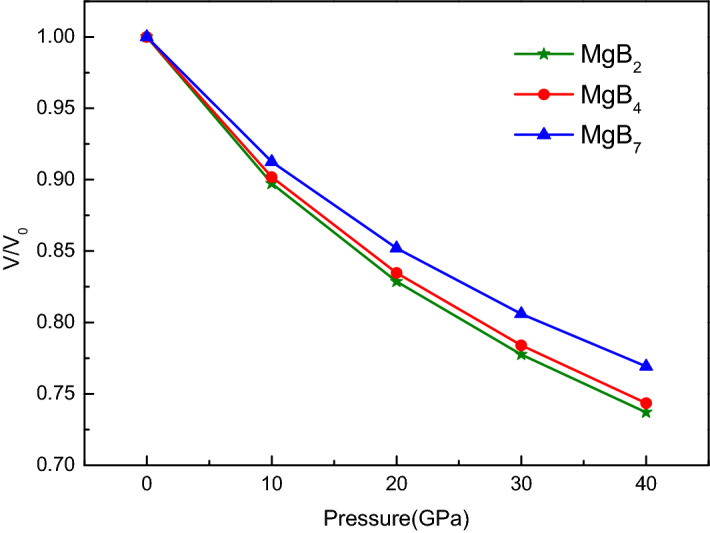
Volume ratio and pressure relation of Mg–B compounds with an interval of 10 GPa.

The functional pressure–volume P(V) data were obtained after the fitting of the E(V) curves to the Birch–Murnaghan model. Therefore, the P(V) curves displayed the relationship between the structural change and the pressure increase with a step of 10 GPa, as shown in Fig. 3. Moreover, the P(V) curves were calculated by using the equilibrium thermodynamic relation as follows^34^:

3 $$ P\left( V \right) = \frac{3}{2}B_{0} \left( {\left( {\frac{V}{{V_{0} }}} \right)^{ - 7/ 3} - \left( {\frac{V}{{V_{0} }}} \right)^{- 5/3}} \right)\left( {1 + \frac{3}{4}\left( {B_{0}^{{\prime}} - 4} \right)\left( {\left( {\frac{V}{{V_{0} }}} \right)^{- 2/3} - 1} \right)} \right) $$

The volume ratio V/V_0_ of the three Mg–B compounds decreased with an increase in the pressure, as shown in Fig. 3, which was in agreement with the general rules. Moreover, the value of V/V_0_ of the Mg–B compounds under the same pressure from 10 to 40 GPa ranged in the following order: MgB_2_ < MgB_4_ < MgB_7_. That is, MgB_7_ was harder to compress under the same applied pressure, as it had the highest value of V/V_0_ among the three Mg–B compounds. Furthermore, MgB_2_ was the most sensitive to the pressure–volume relationship.

### Mechanical properties

The elastic constants (C_ij_) of the crystal as an indispensable parameter played an important role in characterising the mechanical behaviours, because it contains a significant mechanical information under various pressures. There were nine (C_11_, C_12_, C_13_, C_22_, C_23_, C_33_, C_44_, C_55_, and C_66_) elastic constants for the orthorhombic crystals of MgB_4_ and MgB_7_, and six elastic constants (C_11_, C_12_, C_13_, C_33_, C_44_, and C_66_) for the hexagonal crystal of MgB_2_. Table 2 shown the simulated elastic constants and other elastic parameters of the Mg–B compounds; they were in agreement with the reference data. Moreover, the mechanical stability criterion of the hexagonal and orthorhombic structures of the Mg–B compounds is listed below:

**Table 2:** Elastic constants C_ij_ (GPa) and elastic moduli B, G, and E (GPa) of Mg–B compounds at 0 GPa along with the reported data.

Phase	Species	C_11_	C_22_	C_33_	C_12_	C_13_	C_23_	C_44_	C_55_	C_66_	B	G	E
MgB_2_	Present	419.8		253.6	53.2	41.5		184.3	64.6		151.7	116.2	276.1
	Cal^29^	438.0		254.3	61.0	41.0		185.1	71.3		157.0	116.9	277.6
MgB_4_	Present	231.4	289.6	493.5	22.8	162.9	41.3	96.8	108.6	173.5	158.3	112.4	272.7
MgB_7_	Present	536.5	530.6	500.3	60.8	42.1	45.1	194.3	221.3	248.7	205.8	225.9	496.2
	Cal^20^	539	527.7	496.9	61.1	42.4	44.8	195.1	220.6	250.1	206.5	226.6	497.7

For the hexagonal (MgB_2_) crystal^35^:

4 $$ C_{44} > 0,C_{11} > \left| {C_{12} } \right|,\left( {C_{11} + 2C_{12} } \right)C_{33} - 2C_{13}^{2} > 0 $$

For the orthorhombic (MgB_4_ and MgB_7_) crystal^36^:

5 $$ \begin{aligned} & C_{ii} > 0,\quad i = 1\sim6,\;\;C_{11} + C_{22} > 2C_{12} ,\;\;C_{11} + C_{33} > 2C_{13} \\ & C_{22} + C_{33} > 2C_{23} \quad C_{11} + C_{22} + C_{33} + 2\left( {C_{12} + C_{13} + C_{23} } \right) > 0 \\ \end{aligned} $$

**Figure 4:**
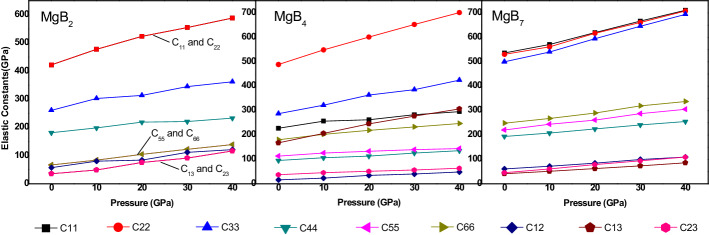
Pressure dependence of the elastic constants of the three Mg–B compounds.

The influence of the applied pressure from 0 to 40 GPa on the calculated elastic constants for the Mg–B compounds is displayed in Fig. 4. As shown in Fig. 4, the entries in the elastic tensor C_ij_ increased with an increase in pressure in the range from 0 to 40 GPa, and when the pressure reached to 40 GPa, all the elastic constants are well matched with the criterion of mechanical stability. Moreover, the deformation resistance of MgB_2_ and MgB_7_ is higher in the x-axis direction than in that of the other axes; this might be attributed to the fact that the largest elastic constant of C_11_ was observed for MgB_2_ and MgB_7_. Similarly, the C_22_ of MgB_4_ with the largest elastic constant indicated that the y-axis had the highest deformation resistance for MgB_4_. According to the existing literature^37^, the values of C_44_, C_55_, and C_66_ are always used to represent the ability of compounds to resist shear deformation. Therefore, MgB_7_ had higher values of C_44_ and C_66_ than the other two Mg–B compounds, according to Fig. 4, which implied that MgB_7_ had the highest resistance ability for the shear deformation.

Generally, the elastic modulus contained B, G, and E could be subsequently obtained by using the VRH (Voigt–Reuss–Hill) approximation^38^, after the elastic constants C_ij_ were obtained. The calculation equations are given by Ref.^39,40^:

6 $$ B_{H} = \frac{1}{2}\left( {B_{V} + B_{R} } \right)\begin{array}{*{20}c} {} & {G_{H} = } \\ \end{array} \frac{1}{2}\left( {G_{V} + G_{R} } \right)\begin{array}{*{20}c} {} & {E = \frac{9BG}{{3B + G}}} \\ \end{array} $$

For the hexagonal crystal:

7 $$ B_{V} = \frac{2}{9}\left( {C_{11} + C_{12} + 2C_{13} + \frac{1}{2}C_{33} } \right) $$

8 $$ G_{V} = \frac{1}{15}\left( {2C_{11} + C_{33} - C_{12} - 2C_{13} } \right) + \frac{1}{5}\left( {2C_{44} + C_{66} } \right) $$

9 $$ B_{R} = \frac{{\left( {C_{11} + C_{12} } \right)C_{33} - 2C_{13}^{2} }}{{C_{11} + C_{12} - 4C_{13} + 2C_{33} }} $$

10 $$ GR = \frac{{5C^{2} C_{44} C_{66} }}{{2\left[ {3B_{V} C_{44} C_{66} + C^{2} \left( {C_{44} + C_{66} } \right)} \right]}} $$

11 $$ C^{2} = \left( {C_{11} + C_{12} } \right)C_{33} - 2C_{13}^{2} $$

For an orthorhombic crystal:

12 $$ B_{V} = \frac{1}{9}\left( {C_{11} + C_{22} + C_{33} + 2\left( {C_{12} + C_{13} + C_{23} } \right)} \right) $$

13 $$ G_{V} = \frac{1}{15}\left[ {C_{11} + C_{22} + C_{33} + 3\left( {C_{44} + C_{55} + C_{66} } \right) - \left( {C_{12} + C_{13} + C_{23} } \right)} \right] $$

14 $$ \begin{aligned} B_{R} & = \Delta /\left[ {C_{11} } \right.\left( {C_{22} + C_{33} - 2C_{33} } \right) + C_{22} \left( {C_{33} - 2C_{13} } \right) - 2C_{33} C_{12} \\ & \quad + C_{12} \left( {2C_{23} - C_{12} } \right) + C_{13} \left( {2C_{12} - C_{13} } \right) + C_{23} \left. {\left( {2C_{13} - C_{23} } \right)} \right] \\ \end{aligned} $$

15 $$ \begin{aligned} G_{R} & = 15\left\{ 4 \right.\left[ {C_{11} \left( {C_{22} + C_{33} + C_{23} } \right)} \right. + C_{22} \left( {C_{33} + C_{13} } \right) + C_{33} C_{12} - C_{12} \left( {C_{23} + C_{12} } \right) \\ & \quad - \left. {C_{13} \left( {C_{12} + C_{13} } \right) - C_{23} \left( {C_{13} + C_{23} } \right)} \right]/\Delta + 3\left( {\frac{1}{{C_{44} }} + \frac{1}{{C_{{{55}}} }} + \frac{1}{{C_{{{66}}} }}} \right) \\ \end{aligned} $$

16 $$ \Delta = C_{13} \left( {C_{12} C_{23} - C_{13} C_{22} } \right) + C_{23} \left( {C_{12} C_{13} - C_{23} C_{11} } \right) + C_{33} \left( {C_{11} C_{22} - C_{12}^{2} } \right) $$

**Figure 5:**
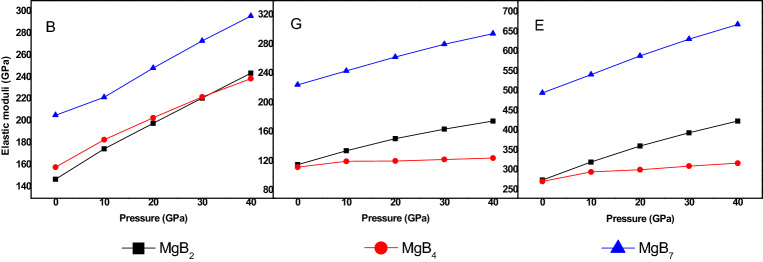
Elastic modulus (B, G, and E) of Mg–B compounds various from different pressure.

As displayed in Fig. 5, the elastic moduli consisting of bulk modulus B, shear modulus G, and Young’s modulus E increased linearly with an increase in the pressure, which indicated that the resistance to the volume deformation increased. From the reports, we inferred that the higher the bulk modulus was, the better the resistance to deformation was Ref.^41^. Simultaneously, we found that MgB_7_ had a stronger capacity to resist volume deformation and had higher hardness, as it had the highest values of the elastic moduli than others at a constant pressure. From Fig. 5, we inferred that the volume deformation resistance ability under a pressure from 0 to 30 GPa deferred to the following increased order: MgB_2_ < MgB_4_ < MgB_7_. Nevertheless, the ability resist to the volume change of MgB_2_ was stronger than others under pressures of 30–40 GPa, and it’s G and E were larger than that of MgB_4_’s. Therefore, it would be inaccurate to predict the hardness of MgB_2_ and MgB_4_.

**Figure 6:**
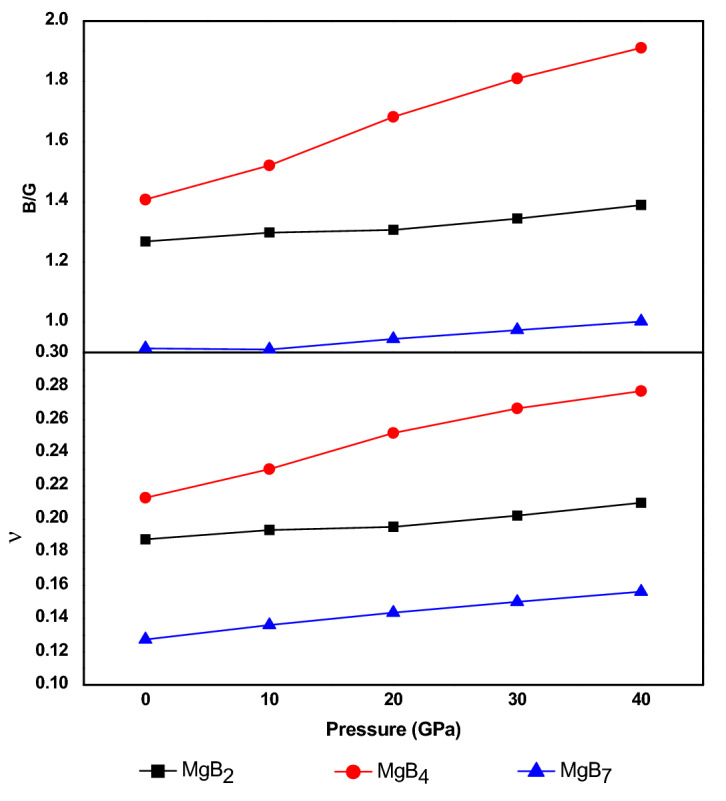
Pressures dependence of the B/G ratio and Poisson’s ratio ʋ.

In general, the B/G ratio and Poisson’s ratio ʋ can be used to explain the ductility and brittleness of Mg–B compounds^42,43^. Figure 6 presents the relation between the B/G ratio and Poisson’s ratio of Mg–B compounds under changed pressure. Poisson’s ratio ʋ can be calculated as follows:

17 $$ \nu = \frac{3B - 2G}{{6B + 2G}} $$

The B/G ratio and ʋ were proposed to describe the brittle or ductile of materials, when the values were 1.75 and 0.26, respectively. As shown in Fig. 6, the Poisson’s ratio ʋ and B/G ratio of MgB_4_ were 0.267 and 1.80 at 30 GPa, and 0.277 and 1.91 at 40 GPa, respectively. Thus, MgB_4_ showed ductile behaviour under pressures of 30–40 GPa, but brittle behaviour at 0–30 GPa, illustrating that the ductile transition for MgB_4_ occurred when the pressure increased to 30 GPa. Nevertheless, MgB_2_ and MgB_7_ displayed a brittle nature, and the brittleness of Mg–B compounds could be ranked in the following order: MgB_4_ < MgB_2_ < MgB_7_. Moreover, all the B/G ratios and ʋ values increased with an increase in the pressure, which indicates that the ductility could be improved by increasing the applied pressure on the Mg–B compounds.

The hardness H is an important parameter to measure the structure and mechanical properties, which can be calculated by using the following semi-empirical law^44^:

18 $$ H = \frac{{\left( {1 - 2\nu } \right)E}}{{6\left( {1 + \nu } \right)}} = \frac{G*E}{{9B}} $$

**Figure 7:**
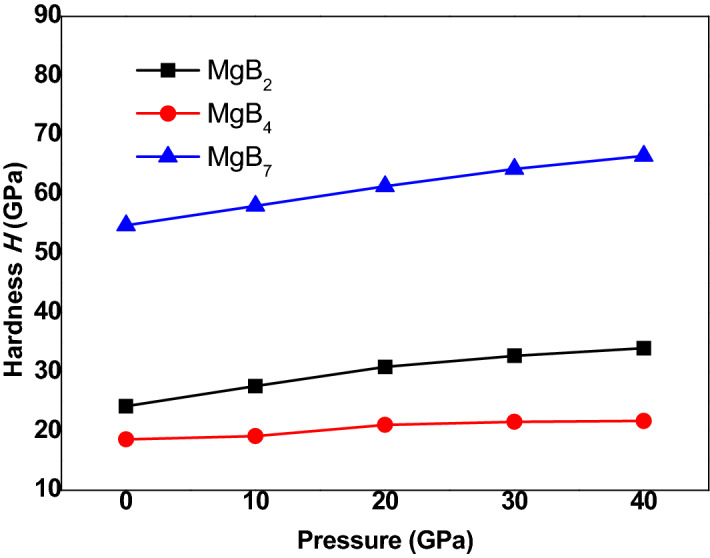
Variation of micro-hardness at various pressures.

As shown in Fig. 7, the hardness of Mg–B compounds increased with an increase in the external pressure, and the values of hardness could be rowed as the following order: MgB_7_ > MgB_2_ > MgB_4_, which implied that MgB_7_ had the highest hardness; this finding matched well with the above mentioned results. On the basis of a comparison with Fig. 5, we can summarized that the effects of G and E on the hardness was greater than B.

Elastic anisotropy plays an important role in crack behaviour and phase transformations, and its formula is defined as follows^45^:

19 $$ A^{U} = 5\frac{{G_{V} }}{{G_{R} }} + \frac{{B_{V} }}{{B_{R} }} - 6 $$

As listed in Table 3, all the predicted values of A_U_ were greater than zero, which implied that all the three Mg–B compounds were anisotropic materials. Moreover, the values of A_U_ of MgB_4_ increased with an increase in the applied pressure, indicating that the anisotropy of MgB_4_ was enhanced by the increase in the applied pressure. Moreover, the elastic anisotropy of MgB_4_ was more sensitive to pressure according to the increase in the values of A_U_, and the anisotropy from low to high could be ranked in the following order: MgB_7_ < MgB_2_ < MgB_4_.

**Table 3:** Universal anisotropy A_U_ of Mg–B binary compounds at external pressure.

Universal anisotropy	Pressure (GPa)	MgB_2_	MgB_4_	MgB_7_
A^U^ (GPa)	0	1.231	1.865	0.153
	10	0.910	1.987	0.131
	20	0.690	2.151	0.118
	30	0.466	2.236	0.108
	40	0.393	2.362	0.104

### Electronic properties

**Figure 8:**
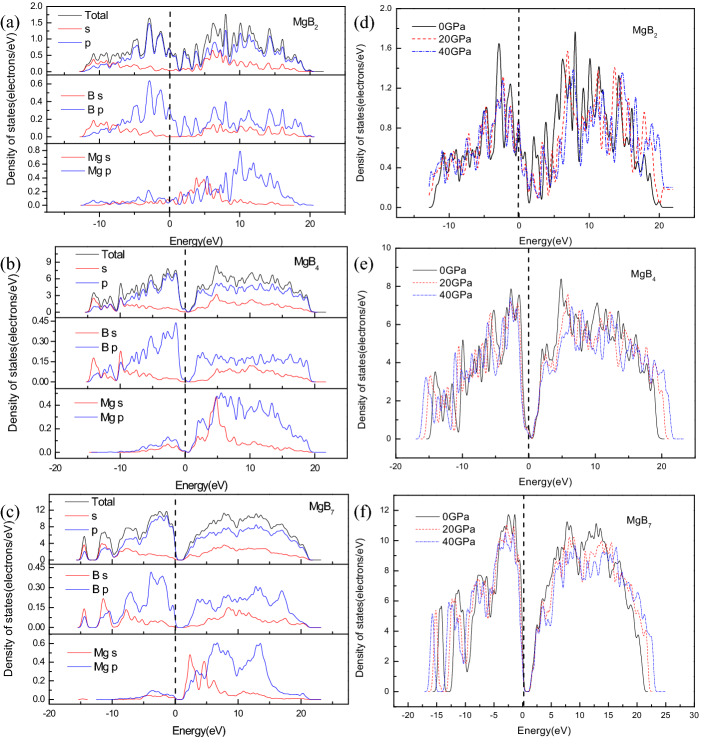
Density of states of Mg–B compounds, (**a**, **b**, **c**) are partial density of states for MgB_2_, MgB_4_, and MgB_7_, respectively; (**d**, **e**, **f**) are total density of states under various pressures for MgB_2_, MgB_4_, and MgB_7_, respectively.

To determine the effects of pressure on the mechanical properties and gain in-depth knowledge of the electronic structure of Mg–B compounds, the partial density of states (PDOS) and the total density of states (TDOS) under various pressures are shown in Fig. 8. Figure 8a,b,c shows that the MgB_2_ presented many peak point near the Fermi level, this indicated that MgB_2_ exhibited its special electrical conductivity, but the Fermi level of MgB_4_ and MgB_7_ are both in the range of zero DOS value, which implied that MgB_4_ and MgB_7_ may present semiconductor or insulator characteristics. Moreover, the primary bond peaks near the Fermi level were mainly occupied by the B 2p states and Mg 3p states for MgB_2_, MgB_4_, and MgB_7_. It can be seen from the Fig. 8, the DOS values of MgB_4_ and MgB_7_ at the Fermi level are all above 0, which implies that both of MgB_4_ and MgB_7_ also present metallic properties. However, for the MgB_7_, the valence band from 2.0 to 5.0 eV, Mg-p band contributes less than the Mg-s band near the Fermi level, The s–p hybridization between the B and Mg atoms forms covalent bonding for MgB_7_. Figure 8d,e,f only depict the TDOS of the Mg–B compounds at 0 GPa, 20 GPa, and 40 GPa, to demonstrate the regularity of TDOS for Mg–B compounds under various pressures. They show that there was a slight decrease in TDOS with an increase in the external pressure, which indicated that there was no structural phase transformation and small interaction potentials changed because of the decrease in the atomic distance under pressure. These figures also display the structural stability and the various electronic characteristics of Mg–B compounds under applied pressure.

### Thermodynamic properties

The quasi-harmonic Debye model of the phonon density of states was implemented in this part of the study to investigate the thermodynamic behaviours of the Mg–B compounds under pressure, namely the heat capacity C_v_, C_p_, the linear thermal expansion coefficient, and the Debye temperature ΘD of the Mg–B compounds. The above-obtained E(V) curves, as important input data for numerical minimisation programs in this model, were used to obtain more thermodynamical information of the Mg–B compounds^46,47^. Moreover, the vibrational thermodynamic properties were obtained at a designated temperature in the quasi-harmonic Debye model; this might be attributed to the consideration of the vibrational contribution for the internal energy. To improve the calculated precision of the thermodynamic behaviours, the 21 volume points from 0.80 a to 1.20 a of the calculated energy–volume were implied.

**Figure 9:**
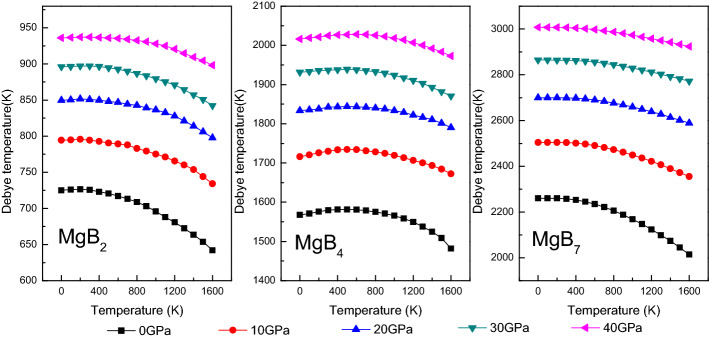
Debye temperature of Mg–B compounds under various pressure and temperature.

The Debye temperature of the Mg–B compounds was calculated from the average sound velocity by using the following formula^48^:

20 $$ \begin{aligned} \Theta D & = \frac{h}{{k_{B} }}\left[ {\frac{3n}{{4\pi }}\left( {\frac{{N_{A} \rho }}{M}} \right)} \right]^{1/3} \nu_{m} \\ \nu_{m} & = \left[ {\frac{1}{3}\left( {\frac{2}{{\nu_{s}^{3} }} + \frac{1}{{\nu_{l}^{3} }}} \right)} \right]^{- 1/3} \quad \nu_{s} = \sqrt {\frac{G}{\rho }} \quad \nu_{l} = \sqrt {\frac{3B + 4G}{{3\rho }}} \\ \end{aligned} $$

where ν_m_, ν_s_, and ν_l_ represent the average wave velocity and the shear and longitudinal sound velocities, respectively; h is Planck’s constant; k_B_ is Boltzmann’s constant; n is the total number of atoms; N_A_ is Avogadro’s number; ρ is the density; and M is the molecular weight. As shown in Fig. 9, the ΘD of the Mg–B compounds increased with an increase in the pressure and remained almost constant from 0 to 200 K but linearly decreased after 200 K. Simultaneously, the ΘD of the Mg–B compounds from low to high could be rowed as the following order: MgB_2_ < MgB_4_ < MgB_7_, when all the compounds were under the same temperature and pressure conditions.

**Figure 10:**
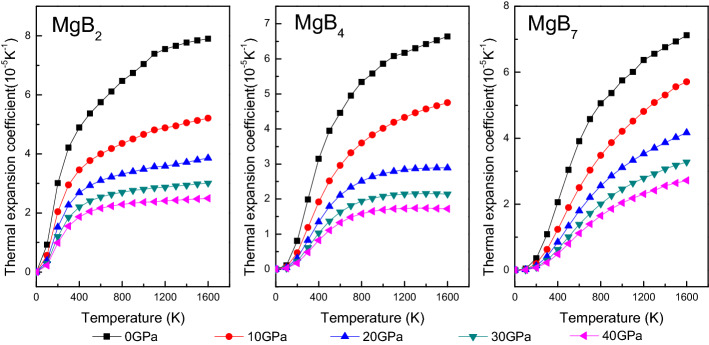
Thermal expansion coefficient of Mg–B compounds as function of pressure and temperature.

Figure 10 shows the temperature and pressure dependence of the volumetric thermal expansion coefficient α of the Mg–B compounds. The thermal expansion coefficient is defined as $\alpha = \frac{1}{{\text{V}}}\frac{{\partial {\text{V}}}}{{\partial {\text{T}}}}$, and the thermal expansion coefficient α increased with an increase in the pressure and the temperature. Although α was linear with T^3^ in the range from 0 to 300 K, α presented a gradual growth rate and changed gently when the temperature exceeded 300 K, which implied that the main thermal expansion of the Mg–B compounds occurred in the low-temperature region. In addition, α presented a decreasing tendency when the pressure increased to 40 GPa at a constant temperature. Meanwhile, the impact strength of pressure on the thermal expansion coefficient increased when the pressure was above 20 GPa.

**Figure 11:**
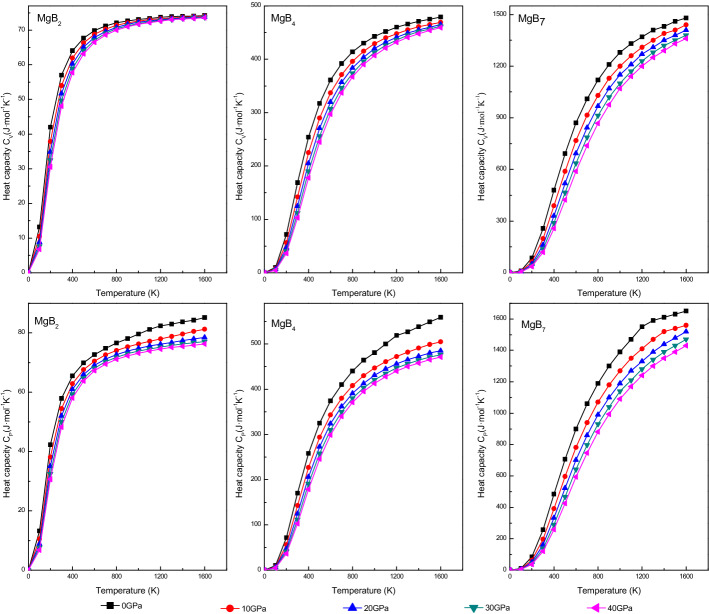
Pressure and temperature dependence of heat capacity C_V_, C_P_ of Mg–B compounds.

The heat capacity are estimated by using Debye temperature and electronic structures of Mg–B compounds, which defined as follows:

21 $$ \begin{aligned} C_{V} &= 3nk\left[ {4D\left( {\frac{\Theta }{T}} \right) - \frac{3\Theta /T}{{e^{\Theta/T} - 1}}} \right]  \\ {\text{C}}_{{\text{P}}} &= \frac{{\pi^{2} {\text{K}}_{{\text{B}}}^{2} {\text{D}}_{{\text{f}}} T}}{3} + \frac{{12\pi^{4} RnT^{3} }}{{5\theta_{D}^{3} }},\quad \gamma = \frac{{\pi^{2} {\text{K}}_{{\text{B}}}^{2} {\text{D}}_{{\text{f}}} }}{3},\quad \beta = \frac{{12\pi^{4} Rn}}{{5\theta_{D}^{3} }}  \end{aligned} $$

where γ and β are the electronic and phonon contributions to the specific heat respectively. The temperature and the pressure dependence of the isochoric heat capacity (C_V_) and the isobaric heat capacity (C_P_) of the Mg–B compounds are displayed in Fig. 11. When the temperature was below 300 K, the variation of C_V_ and C_P_ exhibited an obvious and sharp rise; this subordinated Debye’s law. However, C_P_ and C_V_ were likely to continue to increase and remained constant after 300 K, respectively, due to the C_V_ abided by the Dulong–Petit limit under high temperature conditions. Moreover, both the isochoric heat capacity (C_V_) and the isobaric heat capacity (C_P_) decreased with an increase in the pressure. Thus, from Fig. 11, we inferred that the heat capacity of MgB_7_ was higher than that of MgB_4_ and MgB_2_, indicating the stronger ability of release and absorption energy of MgB_7_.

## Conclusion

In this investigation, the structural, mechanical, electronic, and thermodynamic properties of Mg–B compounds were studied by using density functional theory; the conclusions of this paper can be summarized as follows:

1. The simulated elastic constants and elastic modulus through first-principle method are well coincided with the experimental values and theoretical calculations. The ratio of V/V_0_ decreased with an increase in the external pressure and increased with an increase in the boron content.
2. The three Mg–B compounds are mechanically stable from 0 to 40 GPa. The additional pressure on MgB_2_, MgB_4,_ and MgB_7_ can improve it’s B, G, and E, which rowed as the following order: MgB_2_ < MgB_4_ < MgB_7_, but these properties of MgB_2_ is more excellent than that of MgB_4_ when the pressure reach to 30 GPa. Besides, a ductile conversion behavior at 30GPa is found in the process of increasing the pressure for MgB_4_.
3. The hardness of three Mg–B compounds enhanced with the increased pressure, the hardness of Mg–B compounds could be rowed as following order: MgB_7_ > MgB_2_ > MgB_4_. Conversely, the calculated elastic anisotropy could be ranked as following order: MgB_7_ < MgB_2_ < MgB_4_.
4. From the results the total density of states (TDOS) and the partial density of states (PDOS), the main orbital hybridizations of Mg–B compounds are B p and Mg d orbitals, B p and Mg s orbitals and B p and Mg p orbitals for MgB_2_, MgB_4_ and MgB_7_, respectively. There has no phase transformation under the rising external pressure. From the band structure, the MgB_7_ and the MgB_4_ shows semiconductor properties, but MgB_2_ presents excellent conductivity characteristic.
5. The Debye temperature of all Mg–B compounds reduce with an increase temperature from 0 to 1400 K but increase with an increase pressure from 0 to 40 GPa. The linear thermal expansion coefficient α increase linearly with an increase temperature and pressure, while it present a sharp increase when the pressure is rising up to 40GPa. The results of the isochoric heat capacity (C_V_) and the isobaric heat capacity (C_P_) increase gradually with an increase temperature, while the C_V_ remain unchanged at higher temperature due to followed the Dulong–Petit limit.

## Data Availability

Some or all data, models, or code generated or used during the study are proprietary or confidential in nature and may only be provided with restrictions.
